# Patterns and characteristics of metachronous distant metastases in patients with oral squamous cell carcinoma – A cohort analysis

**DOI:** 10.1007/s00784-026-07057-6

**Published:** 2026-08-12

**Authors:** Jakob C. Roehl, Ralf Smeets, Ann-Kristin Struckmeier

**Affiliations:** 1 Department of Oral and Maxillofacial Surgery, University Medical Center Hamburg-Eppendorf, Martinistraße 52, 20246 Hamburg, Germany; 2 Department of Oral and Maxillofacial Surgery, Division of Regenerative Orofacial Medicine, University Medical Center Hamburg-Eppendorf, Hamburg, Germany

**Keywords:** Distant metastasis, Metachronous, Metastasis, Oral Cancer, Oligometastasis, Squamous cell carcinoma

## Abstract

**Objectives:**

Distant metastases (DMs) are a significant prognostic factor in oral squamous cell carcinoma (OSCC). This study analyzed the pattern and predictors of metachronous DMs (MDMs) over time in a large OSCC cohort treated with curative intent.

**Materials and methods:**

A total of 1,122 patients with primary OSCC were treated at the University Medical Center Hamburg-Eppendorf between 2009 and 2019. After excluding patients with synchronous metastases or palliative treatment, 1,040 patients remained for analysis.

**Results:**

Of these, 93 patients (8.94%) developed MDMs. The lungs were the most frequent site (*n* = 64), followed by bone (*n* = 26), thoracic lymph nodes (*n* = 19), and liver (*n* = 17). The estimated mean time to MDM was approximately 430 days. In patients with late-onset metastasis (≥ 24 months), the lung was most often the sole site involved. Multiple metastatic sites were observed in 40.86% of cases. Cox regression analysis identified several independent factors associated with early metachronous dissemination (≤ 12 months after diagnosis): pN2c nodal status (HR 10.778, 95% CI 1.123–103.405, *p* = 0.039), age < 60 years (HR 3.626, 95% CI 1.048–12.547, *p* = 0.042), and treatment with surgery plus adjuvant radiochemotherapy (HR 20.025, 95% CI 1.954–205.232, *p* = 0.012).

**Conclusion:**

The occurrence of MDMs was observed in around 10% of OSCC patients. Early metachronous dissemination was associated with pN2c, age < 60 years and combination therapy.

**Clinical Relevance:**

These findings support a time- and pattern-dependent model of distant dissemination in OSCC and may inform risk-adapted surveillance and treatment strategies.

**Supplementary Information:**

The online version contains supplementary material available at https://doi.org/10.1007/s00784-026-07057-6.

## Introduction

Oral squamous cell carcinoma (OSCC) is the most prevalent mucosal primary cancer of the mouth [1, 2]. Due to its tendency for locoregional spread, curative treatment typically involves surgical resection with clear margins and neck dissection [3, 4]. Despite advancements in surgical techniques, adjuvant radiotherapy, and systemic therapies, distant metastases (DMs) remain a clinically significant challenge [5–7]. The lungs, liver, and bones are the most frequent sites of DMs in head and neck squamous cell carcinoma (HNSCC) [6–8]. Among patients who develop DMs, including those with OSCC, the prognosis is poor, with median survival in published cohorts typically below twelve months [9–11]. Notably, OSCC-specific survival data after metachronous distant metastasis are sparse, with most prognostic estimates derived from broader HNSCC cohorts — a gap that motivated the present focused analysis. Furthermore, the overall incidence of DMs in OSCC is relatively low – estimated between 10 and 20% – their presence typically marks a terminal phase of the disease [11, 12]. Emerging evidence suggests biological and temporal heterogeneity in patients with DMs. Increasing attention is being given to distinctions such as synchronous versus metachronous DMs, as well as oligometastatic versus polymetastatic states, due to their potential prognostic and therapeutic implications [13, 14]. Metachronous DMs (MDMs), defined as metastases occurring after a disease-free interval following definitive treatment, are particularly relevant in HNSCC. These lesions often present in isolation and may be amenable to metastasis-directed therapies (MDTs) in selected patients [12, 15]. Despite their clinical significance, MDMs remain under-explored. For instance, Hosni et al. examined a cohort of OSCC patients treated with postoperative intensity-modulated radiotherapy (IMRT) and identified the predictive value of nodal stage and histologic grade for distant failure. However, their study did not address the timing or anatomical distribution of metastases, nor did it distinguish between oligometastatic and polymetastatic patterns [12]. Given these knowledge gaps, this study aimed to characterize the anatomical distribution, timing, and prognostic implications of MDMs in OSCC patients treated with curative intent. Additionally, we sought to identify clinicopathological risk factors associated with early and polymetastatic progression.

## Methods

**Fig. 1:**
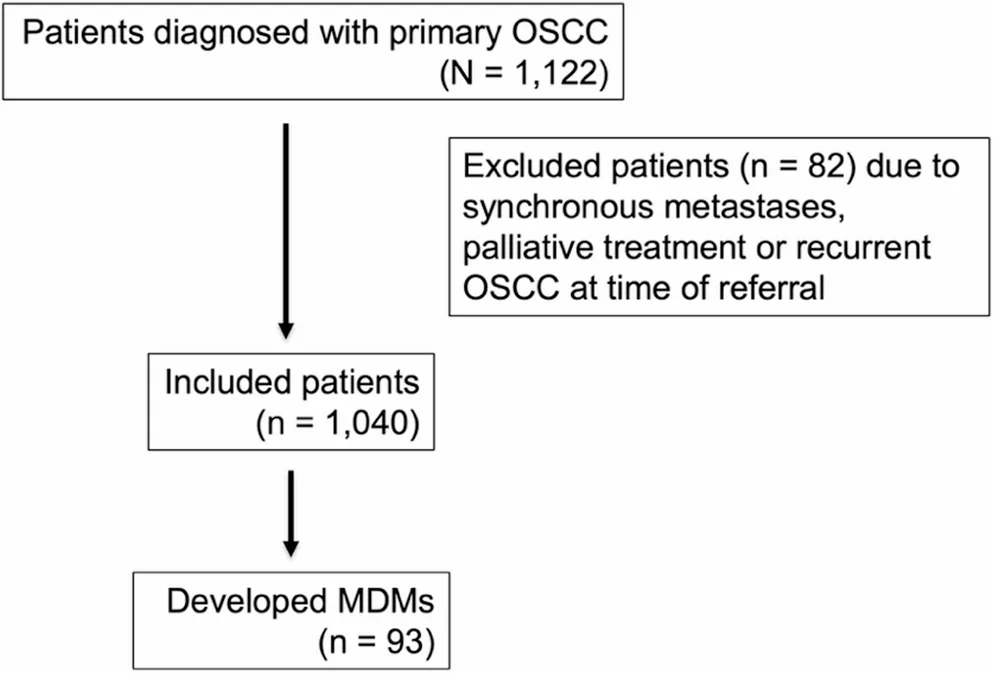
Flow diagram of patient selection

**Fig. 2:**
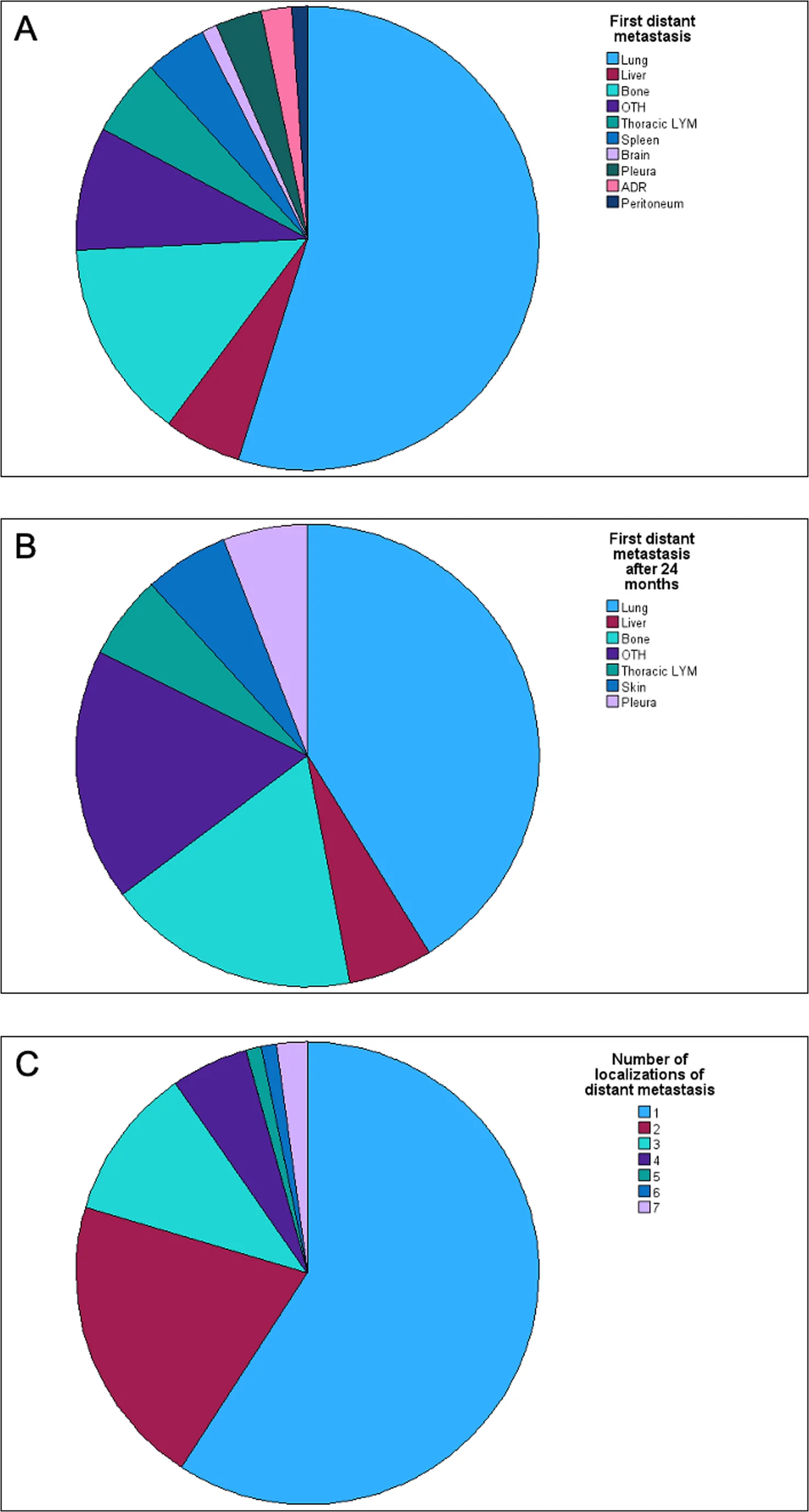
Distribution of metachronous distant metastases in patients with OSCC (*N* = 93). (**A**) Localization of the first metachronous distant metastasis, (**B**) Localization of the first metachronous distant metastasis after 24 months, and (**C**) Number of localizations of metachronous distant metastasis

This retrospective cohort study included 1,122 patients diagnosed with primary OSCC who received primary tumor treatment at a high-volume tertiary center in Germany between January 1, 2009, and December 31, 2019. Of the 1,122 patients initially identified, (*n* = 19) were excluded due to synchronous distant metastasis at presentation, (*n* = 50) due to recurrent OSCC at the time of referral, and (*n* = 32) due to upfront palliative-intent treatment, leaving 1,040 patients eligible for analysis. The patient selection process is illustrated as a CONSORT-style flow diagram in Fig. 1. The last follow-up date was November 2024. The final statistical analysis was performed in December 2024 (Fig. 2).

As part of the staging, all patients underwent a contrast-enhanced computed tomography (CT) scan of the neck and thorax. Treatment modalities included surgery alone, surgery with adjuvant radiotherapy or chemotherapy, and primary radiochemotherapy, all administered according to national (German) treatment guidelines. Therapeutic decisions were made by multidisciplinary tumor boards, considering individual patient risk factors. Surgical resection aimed at clear margins (≥ 5 mm wherever anatomically feasible) was combined with elective or therapeutic neck dissection (selective neck dissection levels I–III for cN0 disease; modified radical or comprehensive neck dissection for cN+ disease). Adjuvant radiotherapy was indicated for pT3/pT4, pN+, perineural or lymphatic invasion, or close margins; concomitant chemoradiotherapy was added for extranodal extension or microscopically positive margins, in accordance with the German S3 guideline (AWMF 007-100OL) and NCCN recommendations effective at the time of treatment [4, 16, 17]. In line with the current guidelines, follow-up consisted of clinical and ultrasound examination every three months for the first two years, every six months in years three to five, and annually thereafter. Cross-sectional imaging (contrast-enhanced CT of the neck and thorax) was performed at six-month intervals during the first two years and whenever clinically indicated thereafter. Histological confirmation of distant metastases was obtained whenever the lesion was technically accessible to biopsy or surgical resection; in a minority of cases (typically multifocal pulmonary or osseous lesions in patients with poor performance status), the diagnosis was based on multimodal imaging (contrast-enhanced CT and/or PET-CT) demonstrating lesions characteristic of metastatic disease together with clinical and radiographic progression.

Exclusion criteria included synchronous DMs, recurrent (local or (loco)regional) OSCC, and patients who received palliative treatment prior to the development of DMs. To ensure the analysis focused on truly metachronous events, only patients who were free of local or regional recurrence at the time of DM diagnosis were included. The absence of locoregional recurrence at the time of MDM diagnosis was confirmed by clinical examination by an experienced head-and-neck surgeon, ultrasound of the neck, and cross-sectional imaging of the head and neck (contrast-enhanced CT or MRI). Locoregional recurrences occurring subsequently (i.e., after the index MDM event) were recorded as outcome variables but did not lead to exclusion.

We based the definition of MDMs on the WHO Classification of Head and Neck Tumors (5th edition, 2024), which classifies metachronous distant metastases as metastases occurring more than 6 months after the initial diagnosis [18]. For analysis, the time from the date of surgical resection (or, for patients receiving primary radiochemotherapy, the start of treatment) to first MDM was used as the principal time variable, and patients were stratified into four intervals: ≤6 months, 7–12 months, 13–24 months, and ≥ 25 months after this time origin. In line with the ESTRO–EORTC consensus, oligometastatic disease was defined as ≤ 3 distant metastatic lesions involving ≤ 2 organ systems, all of which were considered amenable to local ablative therapy at the time of diagnosis; polymetastatic disease was defined as > 3 lesions or involvement of > 2 organ systems [19]. 

The study protocol was approved by the Ethics Committee of the Ärztekammer Hamburg (ethics vote: 2024-101379-BO-ff). Informed consent was not required, in accordance with national and institutional regulations. The manuscript adheres to the STROBE reporting guidelines [20]. 

### Clinicopathological characteristics

Clinicopathological data were extracted from patient medical records, including age, sex, tumor localization, TNM classification, grading, resection margins, and the presence of perineural, vascular, and lymphatic invasion. We also recorded the dates of surgery, last follow-up, local, locoregional and distant recurrence, and death. Depth of invasion (DOI) and extranodal extension (ENE) were abstracted where documented but were too incompletely recorded across the study period to be analysed as structured variables (see Limitations). Where pathology reports used only the combined term ‘lymphovascular invasion’ without separate L and V subcodes, the case was classified as L1 with V not assessable (Vx) to avoid spurious double-counting.

Staging was performed according to the Union for International Cancer Control (UICC) TNM classification, the system in routine clinical use at our institution. During the study period, the TNM classification was updated from the 7th to the 8th edition (with the T- and N-category criteria as finalized in the 2020 UICC revision). Patients initially classified according to the 7th edition were reclassified according to the revised UICC 8th edition (2020) to ensure consistency; because depth of invasion was not consistently documented across the study period, the T category was assigned from the greatest tumour dimension and the documented extent of local invasion where DOI was unavailable (see Limitations) [21]. 

### Statistical analysis

Data analysis was conducted using SPSS software, version 28.0 (SPSS, Chicago, IL, USA). Correlation analysis was performed utilizing the chi-square test. Overall survival (OS) was estimated using the Kaplan-Meier method, and comparisons of survival outcomes between patients undergoing different treatment modalities were analyzed using the log-rank test. OS was defined as the time from surgical resection of OSCC to death from any cause, with data censored at the last follow-up for patients who were still alive. The same time origin was applied uniformly to time-to-MDM and MDM-free survival analyses. For identifying prognostic factors of DM, univariate Cox analysis was performed, followed by a multivariate Cox analysis that incorporated factors showing significance in the univariate analysis.

Figures were generated using SPSS. A p-value of < 0.05 was considered statistically significant.

## Results

### Clinicopathological characteristics

**Fig. 3:**
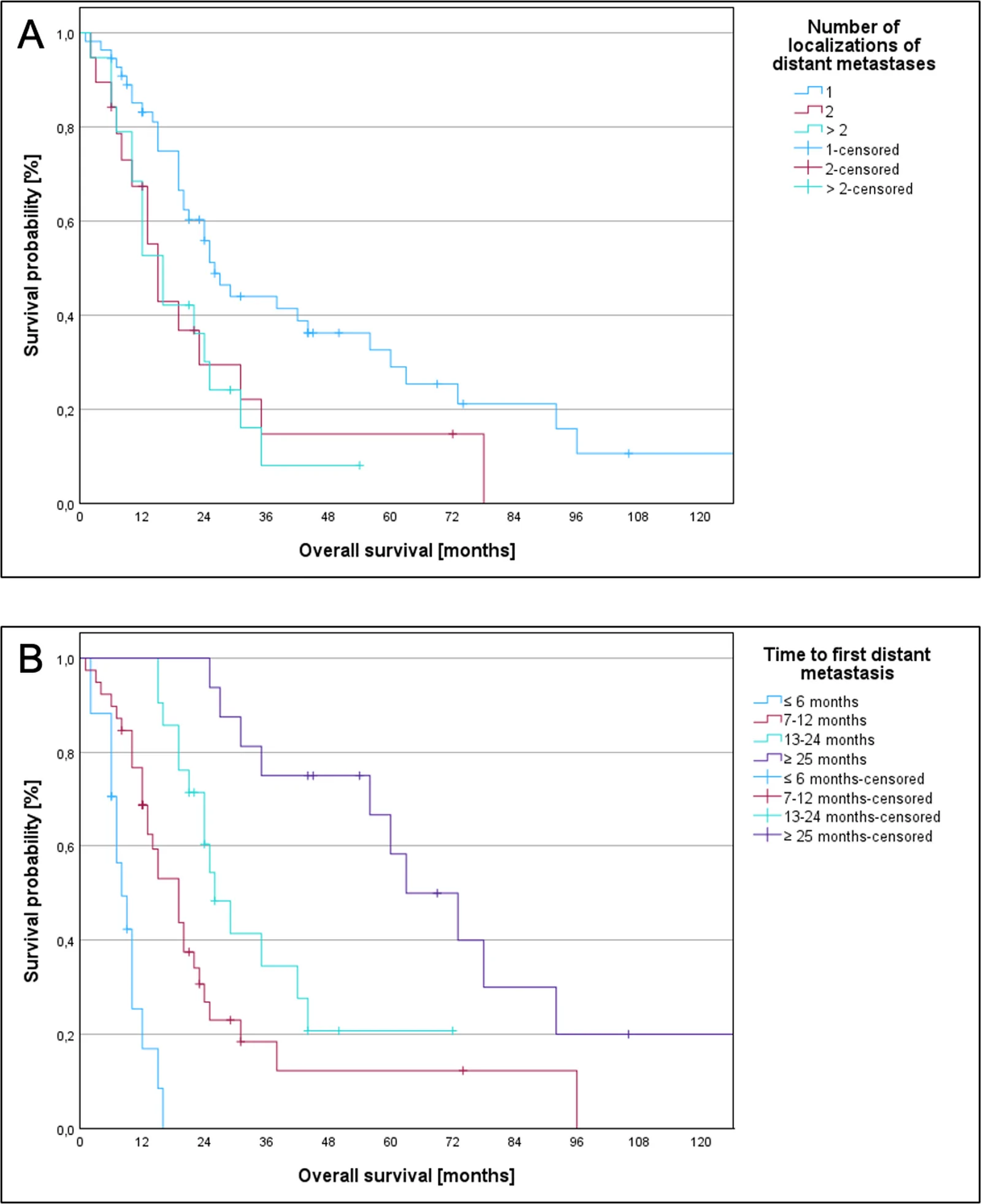
Kaplan-Meier curves of overall survival (OS) of oral squamous cell carcinoma patients with metachronous distant metastases (*n* = 93) depending on (**A**) the number of localizations of distant metastasis and (**B**) the time to first metachronous distant metastasis

The final study cohort comprised 1040 patients who were undergoing treatment for OSCC. In this cohort, 93 patients (93/1040, 8.9%) developed DM. The majority of these patients were male (64/93, 68.2%), with a median age of 60. Of these patients, 47.3% (44/93) were under 60, and 52.7% (49/93) were over 60. The age distribution is depicted in Figure S1. The majority of the tumors leading to DMs were localized either on the floor of the mouth (35/93; 37.6%), the lower jaw (20/93; 21.5%) and the tongue (13/93; 14.0%) (Fig. 3). With regard to the tumor stage, DMs were observed in 21.5% (20/93) of cases in T1, 22.6% (21/93) in T2, and 29.0% (27/93) in T4a. 40.86% (38/93) had no lymph node metastasis (N0) at diagnosis. The distribution of metachronous DMs on pathological T and N stages are depicted in Figures S2 and S3. The majority of tumors exhibited moderate differentiation (G2, 58.1%). Most patients underwent surgery alone (27/93; 29.0%) or surgery plus adjuvant radiation (24/93; 25.8%) (Table 1).

**Table 1:** Clinicopathological characteristics of oral squamous cell carcinoma patients with metachronous distant metastases

Variable	Category	*n* (%)
Sex	Male	64 (68.82)
	Female	29 (31.18)
Age at surgery	< 60 years	44 (47.31)
	≥ 60 years	49 (52.69)
Pathological T stage	T1	20 (21.519
	T2	21 (22.58)
	T3	4 (4.30)
	T4a	27 (29.03)
	Tx	21 (22.58)
Pathological N stage	N0	38 (40.86)
	N1	5 (5.38)
	N2a	4 (4.30)
	N2b	14 (15.05)
	N2c	5 (5.38)
	N3b	6 (6.45)
	Nx	21 (22.58)
M stage at time point of initial therapy	M0	93 (100.00)
Grading	G1	1 (1.08)
	G2	54 (58.06)
	G3	20 (21.51)
	Gx	18 (19.35)
lymphatic invasion	L0	37 (39.78)
	L1	19 (20.43)
	Lx	37 (39.78)
Vascular invasion	V0	43 (46.24)
	V1	12 (12.90)
	Vx	38 (40.86)
Perineural invasion	Pn0	28 (30.11)
	Pn1	17 (18.28)
	Pnx	48 (51.61)
Resection margin	R0	50 (53.76)
	R1	3 (3.23)
	R2	2 (2.15)
	Rx	38 (40.86)
Recurrence	No	74 (79.57)
	Yes	19 (20.43)
Death	No	26 (27.96)
	Yes	67 (72.04)
Localization	Tongue	13 (13.98)
	Floor of the mouth	35 (37.63)
	Lower jaw	20 (21.51)
	Upper jaw	7 (7.53)
	Hard palate	9 (9.68)
	Buccal plane	9 (9.68)
Initial therapy	Surgery only	27 (29.03)
	Surgery + Radiation	24 (25.81)
	Surgery + Radiochemotherapy	17 (18.28)
	Radiochemotherapy	9 (9.68)
	Surgery + Chemotherapy	2 (2.15)
	Not documented	14 (15.05)

### Sites of metachronous distant metastases

Regarding the localization of metastases, lung metastases were detected in 68.8% (64/93) of MDM cases. Bone metastases occurred in 28.0% (26/93), thoracic lymph node metastases in 20.4% (19/93), and liver metastases in 18.3% (17/93). Less common sites included the skin, spleen, brain, pleura, adrenal gland, and peritoneum (Table 2).

**Table 2:** Incidence and localization of metachronous distant metastasis

Localization	Number (%)
Lung	64
Liver	17
Bone	26
OTH	16
Thoracic lymph nodes	19
Spleen	3
Skin	5
Brain	2
Pleura	2
ADR	5
Peritoneum	4

Abbreviations: ADR, Adrenal gland, OTH: other sites (soft tissue)

Multiple metastatic sites per patient were possible; therefore, percentages are not shown as categories are not mutually exclusive

### Timepoint of metachronous distant metastases

The majority of MDMs were diagnosed within 24 months, with a peak incidence between 7 and 12 months. Specifically, 24.7% (23/93) of first MDMs occurred within 6 months of diagnosis, 35.5% (33/93) at 7–12 months, 22.6% (21/93) at 13–24 months, and 17.2% (16/93) at ≥ 25 months.

The timing of metastatic spread was similar across different metastatic sites. Liver metastases appeared at a median of 10 months (mean 13.7 months) after initial treatment, bone metastases at 11 months (mean 14.0 months), and lung metastases at 12 months (mean 21.9 months). Metastases to other distant sites (OTH) developed at a median of 12 months (mean 18.7 months), while thoracic lymph node metastases emerged at a median of 11.5 months (mean 13.7 months). Liver and bone metastases tended to occur slightly earlier than lung metastases. The incidence and localization of first MDMs and first MDMs after 24 months is listed in Table 3.

**Table 3:** Incidence and localization of first metachronous distant metastasis and first metachronous distant metastasis after 24 months

Localization	*n* (%), first metastasis	*n* (%), first metastasis after 24 months
Lung	51 (54.84)	7 (43.75)
Liver	5 (5.38)	1 (6.25)
Bone	13 (13.98)	3 (18.75)
OTH	8 (8.60)	2 (12.50)
Thoracic lymph nodes	5 (5.38)	1 (6.25)
Spleen	4 (4.30)	
Skin	1 (1.08)	1 (6.25)
Brain	3 (3.23)	
Pleura	2 (2.15)	1 (6.25)
ADR	0 (0.00)	
Peritoneum	1 (1.08)	

Abbreviations: ADR, Adrenal gland, OTH: other sites (soft tissue)

Multiple metastatic sites per patient were possible; therefore, percentages are not shown as categories are not mutually exclusive

Notably, the proportion of metastases to less typical sites (e.g., bone or other locations) increased in patients with late-onset MDMs (> 24 months; bone: 14.0% vs. 18.8%; Fig. 2a and b).

### Correlation between clinicopathological characteristics and pulmonary metastasis

No statistically significant associations were found between the occurrence of lung metastases compared to other sites and clinicopathological characteristics, including sex, age, T-stage, N-stage, tumor grading, lymphatic invasion, perineural invasion, resection margin status, recurrence, mortality, initial therapy, or tumor localization (Table 4).

**Table 4:** Association of clinicopathological characteristics with the occurrence of metachronous lung metastases in oral squamous cell carcinoma patients

Variable	Characteristic			
		Other metastases	Lung metastases	Chi^2^
Sex	Male	21 (32.81)	43 (67.19)	0.614
	Female	8 (27.59)	21 (72.41)	
Age at surgery	< 60 years	17 (38.64)	27 (61.36)	0.141
	≥ 60 years	12 (24.49)	37 (75.51)	
Pathological T stage	T1	7 (35.00)	13 (65.00)	0.909
	T2	7 (33.33)	14 (66.67)	
	T3	1 (25.00)	3 (75.00)	
	T4a	11 (40.74)	16 (59.26)	
Pathological N stage	N0	16 (42.11)	22 (57.89	0.414
	N1	1 (20.00)	4 (80.00)	
	N2a	0 (0.00)	4 (100.00)	
	N2b	4 (28.57)	10 (71.43)	
	N2c	3 (60.00)	2 (40.00)	
	N3b	2 (33.33)	4 (66.67)	
Grading	G1	0 (0.00)	1 (100.00)	0.254
	G2	17 (31.48)	37 (68.52)	
	G3	10 (50.00)	10 (50.00)	
lymphatic invasion	L0	14 (37.84)	23 (62.16)	0.942
	L1	7 (36.84)	12 (63.16)	
Vascular invasion	V0	15 (34.88)	28 (65.12)	0.920
	V1	4 (33.33)	8 (66.67)	
Perineural invasion	Pn0	11 (39.29)	17 (60.71)	0.502
	Pn1	5 (29.41)	12 (70.59)	
Resection margin	R0	20 (40.00)	30 (60.00)	0.513
	R1	1 (33.33)	2 (66.67)	
	R2	0 (0.00)	2 (100.00)	
Recurrence	No	5 (26.32)	14 (73.68)	0.608
	Yes	24 (32.43)	50 (67.57)	
Death	No	10 (38.46)	16 (61.54)	0.345
	Yes	19 (28.36)	48 (71.64)	
Initial Therapy	Surgery	10 (37.04)	17 (62.96)	0.229
	Surgery + Radiation	11 (54.83)	13 (54.17)	
	Surgery + Radiochemotherapy	4 (23.53)	13 (76.47)	
	Radiochemotherapy	1 (11.11)	8 (88.89)	
	Surgery + Chemotherapy	0 (0.00)	2 (100.00)	
Localization	Tongue	2 (15.38)	11 (84.62)	0.127
	Floor of the mouth	10 (28.57)	25 (71.43)	
	Lower jaw	11 (55.00)	9 (45.00)	

### Risk factors associated with earlier time to metastasis

In the univariate Cox regression analysis assessing the risk of developing DMSs, several factors emerged as significant prognostic indicators. Notably, the occurrence of locoregional recurrence was significantly associated with a lower risk of early DM (HR: 0.195, 95% CI: 0.066–0.575, *p* = 0.003). Additionally, pN2c nodal status was identified as a significant risk factor (HR: 12.750, 95% CI: 1.613–100.789, *p* = 0.016). In the multivariate analysis, pN2c remained an independent predictor of early DM (HR: 10.778, 95% CI: 1.123–103.405, *p* = 0.039) (Table 5).

**Table 5:** Univariate and multivariate analysis of the risk factors associated occurrence of metachronous distant metastases within (a) 6, (b) 12, and (c) 24 months

Clinicopathological characteristics	Univariate analysis			Multivariate analysis		
	HR	95% CI	*p* value	HR	95% CI	*p* value
Age < 60 vs. ≥ 60 years	0.622	0.253–1.730	0.400			
Sex: male vs. female	1.000	0.359–2.789	1.000			
Pathological T stage						
T1	1					
T2	0.941	0.201–4.412	0.939			
T3	0.000	0.000	0.999			
T4a	1.200	0.288–4.993	0.802			
Pathological N stage						
pN0	1			1		
pN1	5.667	0.717–44.795	0.100	8.125	0.830-79.503	0.072
pN2a	2.833	0.253–34.141	0.412	2.763	0.195–39.093	0.452
pN2b	1.545	0.248–9.619	0.641	1.363	0.173–10.714	0.769
pN2c	12.750	1.613-100.789	0.016*	10.778	1.123-103.405	0.039*
pN3b	4.250	0.582–31.047	0.154	3.002	0.272–33.095	0.369
Grading	2.148	0.715–6.449	0.173			
lymphatic invasion	2.143	0.596–7.703	0.243			
Vascular invasion	1.067	0.241–4.719	0.932			
Perineural invasion	2.000	0.522–7.663	0.312			
Resection margin	2.600	0.702–9.632	0.153			
Initial therapy regime						
Surgery	1					
Surgery + Radiatio	1.567	0.384–5.603	0.575			
Surgery + Radiochemotherapy	2.000	0.476–8.403	0.344			
Recurrence	0.195	0.066–0.575	0.003*	0.265	0.033–2.115	0.210
Localization: lowe jaw/floor of the mouth/tongue vs. upper jaw/Hard palate/buccal plane	1.663	0.601–4.598	0.327			

A p value < 0.05 was considered statistically significant. Statistically significant differences are marked with an asterisk

**Table 6:** Univariate and multivariate analysis of the risk factors associated occurrence of metachronous distant metastases within (a) 6, (b) 12, and (c) 24 months

Clinicopathological characteristics	Univariate analysis			Multivariate analysis		
	HR	95% CI	p value	HR	95% CI	p value
Age < 60 vs. ≥ 60 years	2.619	1.107-6.197	0.028*	3.626	1.048-12.547	0.042*
Sex: male vs. female	1.250	0.506-3.085	0.628			
Pathological T stage						
T1	1			1		
T2	1.364	0.395-4.705	0.624	1.410	0.308-6.463	0.658
T3	4.500	0.395-51.298	0.226	1.856	0.115-29.865	0.662
T4a	5.000	1.393-17.943	0.014*	1.575	0.282-8.804	0.605
Pathological N stage						
pN0	1					
pN1	5.500	0.560-53.986	0.143			
pN2a	1.000	0.000	0.999			
pN2b	1.604	0.452-5.692	0.465			
pN2c	5.500	0.560-53.986	0.143			
pN3b	1.000	0.000	0.999			
Grading	1.372	0.468-4.022	0.564			
lymphatic invasion	1.773	0.522-6.020	0.359			
Vascular invasion	1.846	0.434-7.850	0.406			
Perineural invasion	0.868	0.243-3.099	0.828			
Resection margin	1.000	0.000	1.000			
Initial therapy regime						
Surgery	1			1		
Surgery + Radiatio	2.500	0.801-7.806	0.115	1.684	0.406-6.979	0.472
Surgery + Radiochemotherapy	18.750	2.158-162.892	0.008*	20.025	1.954-205.232	0.012*
Recurrence	0.342	0.103-1.130	0.078			
Localization: lower jaw/floor of the mouth/tongue vs. upper jaw/ard palate/buccal plane	0.788	0.311-1.995	0.615			

A p value < 0.05 was considered statistically significant. Statistically significant differences are marked with an asterisk

**Table 7:** Univariate and multivariate analysis of the risk factors associated occurrence of metachronous distant metastases within (a) 6, (b) 12, and (c) 24 months

Clinicopathological characteristics	Univariate analysis		
	HR	95% CI	p value
Age < 60 vs. ≥ 60 years	2.667	0.832-8.545	0.099
Sex: male vs. female	0.520	0.135-2.010	0.343
Pathological T stage			
T1	1		
T2	5.115	0.914-28.640	0.063
T3	1.000	0.000	0.999
T4a	6.462	1.168-35.736	0.032*
Pathological N stage			
pN0	1		
pN1	1.000	0.000	0.999
pN2a	1.000	0.000	0.999
pN2b	1.707	0.317-9.177	0.533
pN2c	1.000	0.000	0.999
pN3b	1.000	0.000	0.999
Grading	5.917	0.570-61.398	0.136
lymphatic invasion	0.706	0.107-4.651	0.717
Vascular invasion	1.158	0.117-11.454	0.900
Perineural invasion	1.231	0.103-14.696	0.870
Resection margin	1.000	0.000	0.999
Initial therapy regime			
Surgery	1		
Surgery + Radiatio	1.591	0.337-7.505	0.557
Surgery + Radiochemotherapy	1.000	0.000	0.998
Recurrence	0.953	0.240-3.788	0.946
Localization: lower jaw/floor of the mouth/tongue vs. upper jaw/hard palate/buccal plane	2.981	0.627-14.187	0.170

A p value < 0.05 was considered statistically significant. Statistically significant differences are marked with an asterisk.

Analyses were performed using available-case data. The number of evaluable cases for each variable can be derived from Table 1

In the analysis of DMs within 12 months, age < 60 years (HR: 2.619, 95% CI: 1.107–6.197, *p* = 0.028) and the initial treatment modality of surgery plus adjuvant radiochemotherapy (HR: 18.750, 95% CI: 2.158–162.892, *p* = 0.008) were significant in univariate analysis. Both factors were confirmed in the multivariate model as independent predictors (age: HR: 3.626, 95% CI: 1.048–12.547, *p* = 0.042; treatment: HR: 20.025, 95% CI: 1.954–205.232, *p* = 0.012). T4a tumor stage was also significantly associated with reduced DM-free survival in univariate analysis (HR: 5.000, 95% CI: 1.393–17.943, *p* = 0.014); however, this association was not retained in the multivariate model (Table 6.)

In the univariate analysis of DMs occurring within 24 months, T4a stage remained significant (HR: 6.462, 95% CI: 1.168–35.736, *p* = 0.032) (Table 7.)

### Risk factors associated with the occurrence of multiple distant metastases

Regarding the occurrence of multiple DMs (polymetastatic disease), T2 and T4a tumor stages were both associated with a significantly reduced risk (T2: HR: 0.267, 95% CI: 0.072–0.981, *p* = 0.047; T4a: HR: 0.281, 95% CI: 0.083–0.949, *p* = 0.041) (Table S1a). pN2c status showed a non-significant trend toward increased risk of polymetastatic spread (HR: 7.692, 95% CI: 0.778–76.075, *p* = 0.081).

No significant risk factors were identified for predicting the occurrence of DMs at sites other than the lung or liver (Table S1b).

### Survival analysis

Pairwise survival analysis revealed significantly poorer outcomes in patients with metastases at two sites compared to those with metastases at only one site (log rank, *p* = 0.026), and in patients with more than two metastases sites compared to those with one (log rank, *p* = 0.009). However, no significant difference in survival was observed between patients with two versus more than two metastases sites (log rank, *p* = 0.811). Corresponding Kaplan-Meier survival curves are presented in Fig. 3.

When stratified by the timing of MDM occurrence, overall survival differed significantly among patients with early (≤ 6 months), intermediate (7–12 months), and late (≥ 25 months) onset of first metastasis (Fig. 3).

Furthermore, the anatomical localization of DMs also influenced survival outcomes. Significant differences were observed between patients with metastases confined to the lung or liver versus those with metastases at other sites (log rank, *p* = 0.044).

## Discussion

This study provides a comprehensive evaluation of the incidence, timing, dissemination patterns, and prognostic implications of MDMs in a large, curative-intent cohort of patients with OSCC. The observed incidence of 8.9% aligns with previously reported ranges (5–12%). However, unlike most prior studies, our exclusive focus on metachronous events offers a more precise depiction of post-treatment metastatic dynamics [13, 14, 22–25]. 

In line with existing literature on DMs in HNSCC, the lung emerged as the most frequent and often the initial site of distant spread (68.8%), even in cases with late-onset metastases (> 24 months). This reinforces its role as a primary and often solitary metastatic niche in OSCC [14]. Secondary sites – including bone, thoracic lymph nodes, and liver – were consistent with organ-specific dissemination patterns reported in historical cohorts, indicating a stable metastatic behavior across tumor subtypes [26]. Although most MDMs developed within 12 months, no organ-specific trend distinguished early from late events. However, while liver and bone metastases occurred relatively early (median ~ 10–11 months), lung metastases, while still the most common, had a wide temporal distribution (median ~ 12 months; mean ~ 22 months). Notably, the proportion of less typical metastatic sites (e.g., bone or other locations) increased in patients with late-onset MDMs (> 24 months), which may have implications for long-term imaging strategies.

Our findings extend the observations of Hosni et al., who identified the lungs as the most common site of distant-only failure following IMRT in OSCC patients, and emphasized the prognostic value of nodal status and histologic grade [12]. However, their cohort included both synchronous and MDMs and did not stratify based on timing or oligometastatic versus polymetastatic progression. In contrast, our study focuses exclusively on MDMs, enabling refined prognostic modeling and potential applications for risk-adapted surveillance and metastasis-directed therapeutic approaches. For instance, the delayed emergence of metastases in atypical locations such as bone underscores the need to tailor follow-up intervals and modalities.

In general, ENE, advanced nodal stage (pN2b/N3), and perineural invasion are known predictors of locoregional recurrence or DMs [7, 24, 27]. In our analysis, we attempted to investigate risk factors based on the localization of DMs, especially in the lungs, but we were unable to identify any predictors. This discrepancy may be partly attributed to our exclusion of patients with synchronous metastases, which likely led to a study population skewed toward tumors with less aggressive baseline behavior. As a result, the metastases observed may reflect delayed dissemination rather than inherently aggressive tumor biology [14]. Despite the absence of predictors for the localization of MDMs, several variables were found to be associated with early dissemination (≤ 12 months). Notably, pN2c status was significantly linked to metastasis within six months, highlighting the prognostic importance of extensive nodal involvement. This aligns with the findings of Hosni et al., who reported that 13% of patients with high-risk OSCC developed DMs after adjuvant chemoradiotherapy, with the strongest correlation observed in pN2 + cases [12]. 

Additionally, the association between nodal burden and early distant spread is well supported in the literature [28–31]. The link between aggressive multimodal therapy and early MDMs in our cohort may reflect confounding by indication, where high-risk patients are more likely to receive intensive treatment. Thus, the therapy itself may not directly promote metastasis, but rather serve as a surrogate for underlying tumor aggressiveness.

The pattern of metastatic dissemination had a pronounced impact on prognosis in our study. Patients with polymetastatic disease exhibited significantly shorter metastasis-free survival compared to those with a solitary metastasis, lending support to the concept of oligometastasis as a distinct and potentially controllable disease state [7, 13, 14]. This aligns with the evolving recognition of oligometastatic cancer as a clinically relevant intermediate entity with therapeutic implications. Additionally, the presence of metastases at atypical sites – outside the lung and liver – was associated with markedly worse survival outcomes, consistent with recent observations in HNSCC and other solid malignancies [7, 14, 22]. These findings underscore the prognostic heterogeneity of DMs and may inform risk stratification and surveillance strategies. The potential benefit of MDT in select HNSCC patients with oligometastatic lung disease has been previously reported [23, 25, 26]. Our data support this approach, particularly for patients presenting with late-onset, isolated pulmonary metastases. In such cases, MDT may not only prolong survival but also delay the need for systemic therapy, especially given the observed stabilization in metastatic burden among long-term survivors.

Our study establishes a statistically significant association between the timing of MDMs and overall survival, thereby refuting the notion that time to first metastasis and survival outcomes are independent. This finding is in accordance with Haring et al., who reported that over 70% of recurrences occurred within the first 12 months following primary treatment, with early recurrences – especially those involving distant spread – being significantly associated with poorer prognosis [22]. These findings advocate for a risk-adapted surveillance model, where follow-up intensity is stratified according to recurrence timing. Moreover, our results reinforce the concept that early dissemination is not a stochastic process but rather may reflect an intrinsic systemic progression pattern present at the time of diagnosis. Taken together, these findings support the interpretation of time to first metastasis as a biologically meaningful variable with direct prognostic and therapeutic implications. Early metastases likely represent a distinct and more aggressive disease phenotype, whereas later-onset MDMs may arise from previously dormant micrometastatic clones or reflect altered tumor-immune interactions over time. Understanding this distinction is vital in guiding treatment decisions – particularly regarding the potential utility of MDT, or the implementation of intensified systemic surveillance in high-risk patient subgroups [5, 7, 13]. While the prognostic implications of synchronous versus metachronous metastasis are well established in head and neck malignancies, the relevance of this distinction in non-HNSCC tumor entities remains debated [32]. 

Furthermore, our results suggest that even with guideline-concordant treatment, specifically adherence to standardized neck dissection protocols, certain patients remain at significant risk for early systemic failure [3, 7, 14, 33]. These findings emphasize that traditional locoregional control strategies may not fully address the risk posed by occult micrometastatic disease in high-risk individuals. While our data confirm the prognostic importance of nodal involvement, they also underscore the limitations of current staging and treatment paradigms in fully capturing systemic risk. Future research may benefit from integrating molecular or immune-based markers to enhance risk stratification and guide adjuvant treatment decisions. Furthermore, the predominance and early occurrence of pulmonary metastases in OSCC highlight the need for vigilant surveillance strategies. Given that lung metastases were observed even in the absence of conventional high-risk features, routine chest imaging – preferably via CT – should be incorporated into standard follow-up protocols for all OSCC patients. This proactive approach may enable earlier detection of distant disease and facilitate timely intervention.

Recent studies have identified the neutrophil-to-lymphocyte ratio (NLR) and other immune-related markers as promising candidates for prognostic assessment [22, 34]. These emerging biomarkers could complement existing staging systems, helping to personalize surveillance intensity and therapeutic strategies. By incorporating immunological or molecular indicators into clinical workflows, we can improve the early detection of metastatic progression, inform treatment decisions, and ultimately enhance patient outcomes. These findings should be interpreted in the context of the recently reported KEYNOTE-689 trial, in which perioperative pembrolizumab in resectable, locally advanced HNSCC – including OSCC – significantly improved event-free survival in patients with PD-L1 combined positive score ≥ 1, with the benefit driven predominantly by a reduction in distant metastatic events [35–37]. Notably, subgroup analyses indicate that the EFS benefit was driven primarily by a reduction in distant metastatic events rather than by improved locoregional control. This is consistent with our observation that early metachronous dissemination – particularly in patients with pN2c disease and those receiving high-intensity multimodal therapy as a marker of underlying tumor aggressiveness – appears to be incompletely controlled by current locoregional treatment. Our data therefore support the rationale for integrating perioperative immunotherapy into the standard-of-care algorithm for high-risk OSCC and identify the patient subgroups (high nodal burden, multimodal-therapy candidates, age < 60 years) most likely to benefit from such intensification. A further conceptual limitation concerns the operational definition of “metachronous”. The WHO-recommended six-month cutoff is a pragmatic rather than a biological discriminator: a proportion of “early” MDMs (in particular those occurring at 6–12 months) likely represent micrometastatic disease that was already present but radiologically undetectable at initial staging. This is consistent with the very high hazard ratios we observed for pN2c disease and combined-modality therapy in the ≤ 6- and ≤ 12-month strata, both of which are biologically plausible markers of subclinical systemic disease at presentation. Future studies incorporating sensitive blood-based biomarkers (e.g., circulating tumor DNA) at baseline will be required to refine the boundary between truly synchronous and truly metachronous events.

### Limitations

This study has several limitations that warrant consideration. First, although the overall cohort was large, the absolute number of patients who developed metachronous distant metastases was small, which results in wide confidence intervals and unstable hazard ratio estimates. Nevertheless, to the best of our knowledge, we are the first group to investigate metachronous metastasis in oral cavity carcinoma in this manner. Secondly, an unavoidable proportion of pathologically unassessable categories (Tx, Nx, Gx, Lx, Vx, Pnx, Rx) is present for several variables, reflecting the heterogeneity of an eleven-year retrospective cohort that includes patients treated with primary (chemo)radiotherapy without prior resection. For the small subset of patients receiving primary radiochemotherapy, the date of treatment initiation rather than surgical resection was used as the time origin. While this allows inclusion of all curative-intent patients, the use of two different anchoring events introduces a minor heterogeneity that should be considered when interpreting the time-to-event analyses. Furthermore, the millimetre margin distance was not consistently documented for the entire cohort, which precluded analysis of the prognostically relevant < 5 mm “close margin” subgroup; only the residual-disease (R) classification was uniformly available. The depth of invasion was not recorded for a subset of older cases, and where only the combined term “lymphovascular invasion” was reported in older pathology reports, the result was classified as L1 with V not assessable, with the possibility of misclassification. Likewise, although extranodal extension (ENE) is a major adverse pathological feature and has constituted an established indication for adjuvant chemoradiotherapy since the RTOG 9501 and EORTC 22,931 trials and the corresponding NCCN recommendations – and was accordingly considered in our multidisciplinary tumour-board decisions throughout the study period – it could not be analysed as a structured variable in the present cohort. In the earlier years of the study period ENE was recorded predominantly in free-text form, with heterogeneous terminology and without a consistent micro-/macroscopic distinction, and it was only formally integrated as a pN modifier with the 8th edition. We therefore acknowledge the absence of ENE and DOI from the multivariable analyses as a relevant limitation. These reductions in completeness had a detrimental effect on the statistical power of some subgroup analyses and the exploratory analysis of pulmonary versus non-pulmonary first MDMs (see Table 5). It is therefore recommended that these results be regarded as hypothesis-generating. Thirdly, in some patients with multifocal disease and limited performance status, MDM diagnosis was based on multimodal imaging rather than histological confirmation. Fourthly, all included patients were treated with curative intent – patients receiving upfront palliative treatment were excluded by design – so that the present series constitutes, by definition, a curative-intent cohort. Within this framework the cohort was deliberately not restricted to fully guideline-adherent treatment and was therefore heterogeneous with respect to treatment modality, including a small number of patients who received primary radiochemotherapy or who, owing to incomplete or non-guideline-adherent treatment courses, did not receive fully guideline-concordant adjuvant therapy. We defined the cohort by curative treatment intent rather than by guideline adherence in order to reflect real-world clinical practice and to avoid additional selection bias, and treatment modality was included as a covariate in the analyses.

Additionally, the lack of standardized definitions for synchronous versus metachronous metastases across the literature complicates direct comparisons with other studies in this domain. Our exclusive focus on MDMs offers a more homogeneous population but may reduce generalizability to broader HNSCC cohorts.

Importantly, the absence of molecular or genomic data is a key limitation. As molecular profiling was not routinely performed and biospecimen availability was restricted, the study could not investigate potential biological or immunological predictors of metastatic spread. Future prospective studies incorporating molecular analyses are needed to validate clinical risk factors and identify novel biomarkers that could improve early detection and risk stratification for systemic dissemination.

## Conclusions

MDMs occurred in approximately 10% of OSCC patients treated with curative intent. Polymetastatic disease was significantly associated with poorer survival outcomes compared to oligometastatic spread, supporting the concept of oligometastasis as a distinct and potentially controllable disease state. Importantly, time to first metastasis emerged as a key prognostic factor, with earlier onset correlating with worse outcomes. Several independent clinical predictors – namely pN2c nodal status, age < 60 years, and combined surgical and radiochemotherapy treatment—were identified as significant risk factors for early metachronous dissemination.

The lung was confirmed as the most common site of metastasis, even in late-onset cases. However, conventional clinicopathological parameters did not predict the occurrence of pulmonary metastases compared to other sites, underscoring the limitations of current risk models. These findings underscore the need for refined, risk-adapted surveillance strategies and emphasize the urgency of integrating molecular and immunological biomarkers into clinical practice to enhance risk stratification and guide individualized post-treatment follow-up.

Supplemental to: Patterns and characteristics of metachronous distant metastases in patients with oral squamous cell carcinoma – A cohort analysis.

Jakob C. Roehl, Ralf Smeets, Ann-Kristin Struckmeier.

## Electronic Supplementary Material

Below is the link to the electronic supplementary material.

Supplementary Material 1

## Data Availability

No datasets were generated or analysed during the current study.

## Abbreviations

ADR: Adrenal gland
DFS: Disease-Free Survival
DM: Distant Metastasis
DOF: Distant-Only Failure
ENE: Extranodal Extension
HNSCC: Head and Neck Squamous Cell Carcinoma
IMRT: Intensity-Modulated Radiotherapy
MDM: Metachronous Distant Metastasis
MDT: Metastasis-Directed Therapy
NLR: Neutrophil-to-Lymphocyte Ratio
OS: Overall Survival
OSCC: Oral Squamous Cell Carcinoma
OT: Other (non-lung/liver/bone/thoracic lymph node) metastatic sites, OTH: other sites (soft tissue)
