# Supplementary material for: Patterns and characteristics of metachronous distant metastases in patients with oral squamous cell carcinoma – A cohort analysis
Source: Clin Oral Investig. 2026 Aug 12;30(9):385. doi: 10.1007/s00784-026-07057-6 (PMC13468846; doi:10.1007/s00784-026-07057-6)
Supplement: Supplementary file 1 [file 784_2026_7057_MOESM1_ESM.docx]

Table S1: Univariate analysis of the risk factors for occurrence of more than one distant metastasis (a) and distant metastasis other than lung or liver (b)

a.

| Clinicopathological characteristics | Univariate analysis | | |
| --- | --- | --- | --- |
|  | HR | 95% CI | p value |
| Age < 60 vs. ≥ 60 years | 0.582 | 0.253-1.340 | 0.203 |
| Sex: male vs. female | 1.194 | 0.486-2.935 | 0.699 |
| Pathological T stage | | | |
| T1 | 1 | | |
| T2 | 0.267 | 0.072-0.981 | 0.047* |
| T3 | 0.667 | 0.077-5.749 | 0.712 |
| T4a | 0.281 | 0.083-0.949 | 0.041* |
| Pathological N stage | | | |
| pN0 | 1 | | |
| pN1 | 0.000 | 0.000 | 0.999 |
| pN2a | 5.769 | 0.545-61.121 | 0.146 |
| pN2b | 1.442 | 0.412-5.048 | 0.567 |
| pN2c | 7.692 | 0.778-76.075 | 0.081 |
| pN3b | 0.962 | 0.155-5.964 | 0.966 |
| Grading | 1.252 | 0.469-3.346 | 0.654 |
| lymphatic invasion | 0.856 | 0.280-2.614 | 0.784 |
| Vascular invasion | 0.821 | 0.225-2.998 | 0.766 |
| Perineural invasion | 0.727 | 0.209-2.525 | 0.616 |
| Resection margin | 0.347 | 0.053-2.257 | 0.268 |
| Initial therapy regime | | | |
| Surgery | 1 | | |
| Surgery + Radiatio | 0.700 | 0.216-2.271 | 0.552 |
| Surgery + Radiochemotherapy | 1.912 | 0.558-6.554 | 0.302 |
| Recurrence | 1.236 | 0.437-3.498 | 0.690 |
| Localization: lower jaw/floor of the mouth/tongue vs. upper jaw/hard palate/buccal plane | 1.491 | 0.592-3.579 | 0.397 |

A p value < 0.05 was considered statistically significant. Statistically significant differences are marked with an asterisk.

b.

| Clinicopathological characteristics | Univariate analysis | | |
| --- | --- | --- | --- |
|  | HR | 95% CI | p value |
| Age < 60 vs. ≥ 60 years | 0.477 | 0.209-1.093 | 0.080 |
| Sex: male vs. female | 0.717 | 0.297-1.731 | 0.459 |
| Pathological T stage | | | |
| T1 | 1 | | |
| T2 | 0.614 | 0.179-2.108 | 0.438 |
| T3 | 0.818 | 0.095-7.016 | 0.855 |
| T4a | 1.023 | 0.320-3.272 | 0.970 |
| Pathological N stage | | | |
| pN0 | 1 | | |
| pN1 | 0.000 | 0.000 | 0.999 |
| pN2a | 3.333 | 0.318-34.989 | 0.316 |
| pN2b | 1.481 | 0.431-5.095 | 0.533 |
| pN2c | 4.444 | 0.454-43.537 | 0.200 |
| pN3b | 2.222 | 0.363-13.618 | 0.388 |
| Grading | 2.086 | 0.751-5.796 | 0.158 |
| lymphatic invasion | 1.306 | 0.419-4.070 | 0.645 |
| Vascular invasion | 2.609 | 0.620-10.983 | 0.191 |
| Perineural invasion | 1.238 | 0.366-4.187 | 0.731 |
| Resection margin | 0.215 | 0.030-1.533 | 0.125 |
| Initial therapy regime | | | |
| Surgery | 1 | | |
| Surgery + Radiatio | 1.077 | 0.358-3.235 | 0.895 |
| Surgery + Radiochemotherapy | 1.538 | 0.452-5.242 | 0.10 |
| Recurrence | 1.910 | 0.677-5.393 | 0.222 |
| Localization: lower jaw/floor of the mouth/tongue vs. upper jaw/hard palate/buccal plane | 1.432 | 0.569-3.601 | 0.446 |

Figure S1: Age distribution in oral squamous cell carcinoma patients with metachronous distant metastasis

Figure S2: Distribution of pathological T stages in oral squamous cell carcinoma patients with metachronous distant metastasis

Figure S3: Distribution of pathological N stages in oral squamous cell carcinoma patients with metachronous distant metastasis
